# SARS-CoV-2 viral genes Nsp6, Nsp8, and M compromise cellular ATP levels to impair survival and function of human pluripotent stem cell-derived cardiomyocytes

**DOI:** 10.1186/s13287-023-03485-3

**Published:** 2023-09-13

**Authors:** Juli Liu, Shiyong Wu, Yucheng Zhang, Cheng Wang, Sheng Liu, Jun Wan, Lei Yang

**Affiliations:** 1 Department of Pediatrics, Indiana University School of Medicine, Herman B Wells Center for Pediatric Research, Indianapolis, IN 46202 USA; 2 Department of Medical and Molecular Genetics, Indiana University School of Medicine, Indianapolis, IN 46202 USA; 3 Center for Computational Biology and Bioinformatics, Indiana University School of Medicine, Indianapolis, IN 46202 USA; 4 Medical Research Institute, Guangdong Provincial People’s Hospital (Guangdong Academy of Medical Sciences), Southern Medical University, Guangzhou, 510080 Guangdong China

**Keywords:** COVID-19, SARS-CoV-2, Cardiomyocyte, Apoptosis, Cardiac dysfunction, Human pluripotent stem cells

## Abstract

**Background:**

Cardiovascular complications significantly augment the overall COVID-19 mortality, largely due to the susceptibility of human cardiomyocytes (CMs) to SARS-CoV-2 virus. SARS-CoV-2 virus encodes 27 genes, whose specific impacts on CM health are not fully understood. This study elucidates the deleterious effects of SARS-CoV-2 genes Nsp6, M, and Nsp8 on human CMs.

**Methods:**

CMs were derived from human pluripotent stem cells (hPSCs), including human embryonic stem cells and induced pluripotent stem cells, using 2D and 3D differentiation methods. We overexpressed Nsp6, M, or Nsp8 in hPSCs and then applied whole mRNA-seq and mass spectrometry for multi-omics analysis. Co-immunoprecipitation mass spectrometry was utilized to map the protein interaction networks of Nsp6, M, and Nsp8 within host hiPSC-CMs.

**Results:**

Nsp6, Nsp8, and M globally perturb the transcriptome and proteome of hPSC-CMs. SARS-CoV-2 infection and the overexpression of Nsp6, Nsp8, or M coherently upregulated genes associated with apoptosis and immune/inflammation pathways, whereas downregulated genes linked to heart contraction and functions. Global interactome analysis revealed interactions between Nsp6, Nsp8, and M with ATPase subunits. Overexpression of Nsp6, Nsp8, or M significantly reduced cellular ATP levels, markedly increased apoptosis, and compromised Ca^2+^ handling in hPSC-CMs. Importantly, administration of FDA-approved drugs, ivermectin and meclizine, could restore ATP levels, thereby mitigating apoptosis and dysfunction in hPSC-CMs overexpressing Nsp6, Nsp8, or M.

**Conclusion:**

Overall, our findings uncover the extensive damaging effects of Nsp6, Nsp8, and M on hPSC-CMs, underlining the crucial role of ATP homeostasis in CM death and functional abnormalities induced by these SARS-CoV-2 genes, and reveal the potential therapeutic strategies to alleviate these detrimental effects with FDA-approved drugs.

**Supplementary Information:**

The online version contains supplementary material available at 10.1186/s13287-023-03485-3.

## Introduction

Since its emergence in late 2019, the severe acute respiratory syndrome coronavirus 2 (SARS-CoV-2), causative agent of COVID-19, has had an unprecedented global impact on public health [1, 2]. To date, SARS-CoV-2 has infected over 760 million people worldwide, resulting in over 1,112,000 deaths in the USA alone. Although respiratory failure is the primary cause of death by SARS-CoV-2 infection, cardiac complications such as acute myocardial injury, myocarditis, arrhythmias, and sudden death significantly contribute to overall mortality [3–5]. Notably, 8–25% of SARS-CoV-2 patients develop concurrent cardiovascular disorders, and these conditions are especially prevalent among COVID-19-related fatalities [6]. Further, patients with pre-existing cardiovascular conditions demonstrate a heightened mortality rate [7, 8]. Moreover, recent studies reveal persistent cardiovascular risk, including inflammatory myocarditis, right ventricular injury, ventricular arrhythmias, myocardial infarction, and heart failure, even 12 months post-infection [9, 10]. Bailey et al. confirmed the vulnerability of human myocardium to SARS-CoV-2 infection, indicating that the virus can directly infect cardiomyocytes (CMs) in COVID-19 patients with myocarditis [11]. The SARS-CoV-2 virus enters human cells via binding to membrane proteins Angiotensin-Converting Enzyme 2 (ACE2), Transmembrane Serine Protease 2 (TMPRSS2), and Transmembrane Serine Protease 4 (TMPRSS4) [12–14]. The high affinity of the SARS-CoV-2 spike (S) protein for ACE2 renders organs/tissues with high ACE2 expression, such as the lungs, small intestines, testes, kidneys, and heart, primary targets for the virus [15]. Recent studies have leveraged CMs derived from human pluripotent stem cells (hPSCs), including human ES cells (hESCs) and iPS cells (hiPSCs), as an in vitro model to examine the pathological effects of SARS-CoV-2 virus on human heart muscle cells [16]. SARS-CoV-2 infection in hPSC-CMs induces increased cell death [17, 18], fractionated sarcomeres [19], abnormal electrical and mechanical functions [20], and inflammation [17], mirroring the CM injuries observed in COVID-19 patients [11].

The SARS-CoV-2 genome encodes up to 27 genes, including Orf9c, a non-structural accessory protein previously shown to induce apoptosis and dysfunction in hPSC-CMs [21]. A comparative analysis of transcriptomic changes triggered by SARS-CoV-2 infection and Orf9c overexpression in hPSC-CMs suggests that other SARS-CoV-2 genes could also contribute to the altered transcriptome of host hPSC-CMs. In this study, we elucidate the extensive deleterious effects of three SARS-CoV-2 genes, Nsp6, Nsp8, and M, on hPSC-CMs. We found their overexpression activated genes enriched in cellular injury and immune signaling pathways while suppressing genes linked to cardiac functional pathways. Overexpression of Nsp6, Nsp8, or M significantly increased apoptosis, and compromised calcium handling of hPSC-CMs. Nsp6, Nsp8, and M could interact with host hPSC-CM proteins, particularly ATPase subunits, leading to a notable reduction in ATP levels. Given the critical roles of ATP in maintaining CM viability and contractility, we investigated pharmaceutical strategies to enhance cellular ATP levels in hPSC-CMs overexpressing Nsp6, Nsp8, or M. We found that two FDA-approved drugs, ivermectin and meclizine, significantly reduced cell death and dysfunction in hPSC-CMs overexpressing these SARS-CoV-2 genes. Overall, our findings demonstrate that SARS-CoV-2 genes Nsp6, Nsp8, and M can extensively alter the transcriptome and proteome of hPSC-CMs, and interact with key ATP production subunits leading to decreased cellular ATP levels. The ATP reduction plays a pivotal role in CM death and abnormalities induced by these SARS-CoV-2 genes, underscoring the potential therapeutic value of enhancing ATP levels.

## Methods

### Human pluripotent stem cell maintenance and cardiomyocyte differentiation

Human embryonic stem cell (hESC) line H9 (WA09, WiCell), and a human induced pluripotent stem cell (hiPSC) line, originally generated from a healthy donor and acquired from Mount Sinai School of Medicine under MTA, were cultured on Matrigel (BD Biosciences, California, USA)-coated plates using mTesR1 medium [22, 23]. For cardiomyocyte differentiation, the monolayer differentiation followed a slightly modified version of the published protocol [24]. Briefly, differentiation was initiated by removing the mTeSR1 medium and adding RPMI1640 (Gibco, Detroit, Michigan, USA) with B27 supplement minus insulin (Gibco) medium containing 6 μM CHIR99021 from day 0 to day 1. Then cells were then induced with RPMI1640/B27 (no insulin) medium from day 1 to day 3, followed by differentiation with RPMI1640/B27 (no insulin) medium containing 5 μM XAV-939 from day 3 to day 5. After day 5, cells were maintained in RPMI1640/B27 (no insulin) medium without any chemicals. Beating cardiomyocytes are typically observed after 7 days of differentiation. For 3D embryoid body (EBs) differentiation, hiPSCs were differentiated toward CMs using our previously established protocol [25, 26]. Briefly, cardiomyocyte differentiation was conducted by forming EBs. EBs were treated with StemPro-34 SFM (1×) medium (Gibco) under the following conditions: days 0–1 with BMP4 (2.5 ng/ml); days 1–4 with BMP4 (10 ng/ml), FGF-2 (5 ng/ml) and Activin A (2 ng/ml); and days 4–20 with XAV939 (5 µM). Beating EBs could be observed on differentiation days 10–13. In the drug treatment assay, all beating EBs were sustained in DMEM medium (no glucose, Gibco) with 10% FBS. Both ivermectin and meclizine were used at final concentrations of 0.5 µM (dissolved in DMSO). The exposure duration for the apoptosis assay was 48 h. All cytokines were from R&D Systems (Minneapolis, Minnesota, USA). All chemicals were from Sigma-Aldrich (Saint Louis, Missouri, USA).

### Lentivirus production and cell transduction

The lentiviral vectors pLVX-EF1alpha-IRES-Puro-2xStrep-GFP, -Nsp6, -Nsp8, M and -Orf9c was transfected into the HEK293T cells (CRL-3216, ATCC, Manassas, Virginia, USA) with the packaging plasmids psPAX2 and pMD2.G (gifts from Dr. Guang Hu in NIH) using X-treme GENE 9 transfection reagent (Roche, Indianapolis, Indiana, USA) according to the manufacturer's instructions. Two days post-transfection, we collected the viral supernatant and removed cellular debris via syringe filtration with a 0.45 μm pore size filter (Millipore, Burlington, Massachusetts, USA). hESCs and hiPSCs, cultured in mTesR1 medium, were incubated with the virus media for 4 h, followed by an overnight culture in fresh mTesR1 medium. The infection process was repeated after 24 h after viral infection, puromycin was added to select puromycin-resistant hESC and hiPSC clones.

### RT-qPCR

RNA was isolated using the RNeasy Kit (Qiagen, Germantown, Maryland, USA). CDNA synthesis was carried out using the High-Capacity RNA-to-cDNA™ Kit (Applied Biosystems, Waltham, Massachusetts, USA). RT-qPCR was performed on QuantStudio 6 Real-Time PCR Systems using Fast SYBR Green Master Mix (Applied Biosystems) according to the manufacturer’s instructions. The RT-qPCR data were normalized to internal control GAPDH or beta-ACTIN employing the 2^−ΔΔCt^ method [27, 28]. The results are presented as the mean ± S.D. from at least three independent experiments. All primer sequences used could be found in Additional file 5: Table S4.

### Western blotting

Proteins were extracted using the Complete™ Lysis-M EDTA-free kit (Roche). Protein samples were mixed with NuPAGE™ LDS Sample Buffer (4 ×) (Thermo Fisher Scientific, Waltham, Massachusetts, USA) and boiled for 5 min at 95 °C. The proteins were then separated on 4–15% Mini-PROTEAN TGX Gels (Bio-Rad, Hercules, California, USA) and transferred to a PVDF membrane utilizing the Trans-Blot® Turbo™ Transfer System (Bio-Rad). The membranes were blocked with 1 × TBST buffer containing 10% non-fat milk and incubated overnight at 4 °C with primary antibody in 1 × TBST buffer containing 5% BSA. Membranes were washed three times for 5 min each with 1 × TTBST buffer, and then incubated for 1 h at room temperature with horseradish-peroxidase-conjugated secondary antibody (Cell Signaling Technology, Danvers, Massachusetts, USA) in a blocking solution composed of 5% BSA in 1 × TBST buffer. Following another three 5-min washes with 1 × TBST buffer, the membranes were developed using the Pierce™ ECL Western Blotting Substrate (Thermo Scientific) and imaged on the ChemiDoc Imaging System (Bio-Rad).

### Flow cytometry

Flow cytometry was performed based on our previous protocol [28]. Briefly, cells were harvested and dissociated by using 0.25% trypsin–EDTA (Corning, Glendale, Arizona, USA) at 37 °C for 10 min. The dissociated cells were fixed in a 4% PFA solution at room temperature for 10 min and washed 3 times with 1 × PBS. For antibody staining, cells were incubated in 1 × blocking PBS buffer (containing 2% goat serum or 5% BSA plus 0.1% saponin) with corresponding primary antibodies at 37 °C for 1 h, followed by secondary antibody staining at 37 °C for 1 h. For TUNEL (Terminal deoxynucleotidyl transferase dUTP nick end labeling) assay, staining was carried out by using the In Situ Cell Death Detection Kit, Fluorescein (Roche) according to the manufacturer's instructions. Flow cytometry analysis was performed on an Attune NxT Flow Cytometer (Thermo Fisher Scientific). Data were analyzed using FlowJo software (Treestar).

### SARS-CoV-2 spike pseudovirus infection

The SARS-CoV-2 Spike pseudotyped lentivirus with GFP reporter (60 concentration, 60×) was purchased from Virongy (Manassas, Virginia, USA). HESC-CMs were cultured in DMEM medium (no glucose) with 10% FBS on a 6-well plate coated with Matrigel. Cells were infected with 1 × or 2 × SARS-CoV-2 Spike pseudotyped lentivirus. After 48 h of infection, flow cytometry was performed to detect GFP^+^ cells. Cells without virus infection served as the blank control cells. Flow cytometry analysis was performed on the Attune NxT Flow Cytometer (Thermo Fisher Scientific), with data analysis performed using FlowJo software (Treestar).

### Apoptosis assay

Live cell apoptotic assay was performed using the Annexin-V-FLUOS Staining Kit (Roche) according to the manufacturer's instructions. Briefly, cells were harvested and dissociated using 0.25% trypsin–EDTA at 37 °C for 10 min. The single cells were then washed in 1 × PBS, resuspended and incubated in 100 µl of Annexin-V-FLUOS labeling solution for 15 min at room temperature, followed by analysis on a BD LSRII cytometer (Becton Dickinson, Franklin Lakes, New Jersey, USA) or Attune NxT Flow Cytometer (Thermo Fisher Scientific). Data were analyzed using FlowJo software (Treestar).

### Intracellular ATP level measurement

The intracellular ATP level was determined using the Luminescent ATP Detection Assay Kit (Abcam, Branford, Connecticut, USA), as per the manufacturer's guidelines. We utilized flow cytometry or a hemocytometer to count live cell numbers, ensuring the same cell number of each group was used for ATP detection. Live cells were resuspended in 50 μl of detergent solution and subjected to shaking at 1200 rpm on an Eppendorf ThermoMixer for 5 min at room temperature. Subsequently, 50 μl of substrate solution was added to the detergent solution, followed by another round of shaking at 1200 rpm on the Eppendorf ThermoMixer for 5 min at room temperature. The entire solution was then transferred to a Nunc™ MicroWell™ 96-Well Flat-Bottom microplate (White Polystyrene Plate, Thermo Scientific). Luminescence detection was performed on a GloMax Discover Microplate Reader (GM3000, Promega, Madison, Wisconsin, USA). For the drug treatment assay, cells were treated for 3 h with a final concentration of both Ivermectin and Meclizine at 0.5 µM.

### Calcium imaging

Cardiomyocytes were adhered to a Matrigel-coated 12-well plate and loaded with X-Rhod-1 (X14210, Invitrogen, Waltham, Massachusetts, USA) in DMEM medium (glucose-free) supplemented with 10% FBS and Pluronic F-127 (final concentration 0.02%, P2443, Sigma-Aldrich) for 15 min at 37 °C. Videos were captured at a rate of 50 frames per second using the All-in-One Fluorescence Microscope BZ-X800 (KEYENCE CORPORATION, Itasca, Illinois, USA). For the drug treatment assay, cardiomyocytes were treated with a final concentration of 0.5 µM ivermectin and meclizine. Calcium handling signals were recorded 1 h post-drug treatment. Video data were analyzed using ImageJ software, employing a custom script to calculate temporal changes in calcium fluorescence intensity.

### Immunofluorescent microscopy

For immunostaining of attached cells, cells were fixed with 4% PFA for 10 min at room temperature. After washing with 1 × PBS, cells were blocked for 1 h with 1 × PBS blocking buffer containing 2% goat serum (or 5% BSA) and 0.1% saponin. The primary antibodies were diluted with blocking buffer and incubated with fixed cells at − 4 °C for overnight. The following day, staining with secondary antibody was conducted, followed by nuclear staining with DAPI. For immunostaining of EBs, live EBs were fixed with 4% PFA for 10 min at room temperature, followed by incubation in 15% sucrose solution at room temperature until all EBs sank. EBs were then embedded in OCT, followed by cryosectioning. The EBs immunostaining protocol was identical to that of the attached cells. For TUNEL experiments, staining of attached cells or EBs sections was carried out using the In Situ Cell Death Detection Kit, Fluorescein (Roche) according to the manufacturer's guidelines. Imaging was acquired using the Leica DM6B imaging system.

### Co-immunoprecipitation (Co-IP)

Co-immunoprecipitation (Co-IP) of the protein samples was performed using the Pierce™ Classic Magnetic IP/Co-IP Kit (Thermo Scientific). For analysis of the Co-IP protein samples, we used Western blotting or submitted the samples to the Proteomics Core Facility at the Indiana University School of Medicine (IUSM) for mass spectrometry analysis.

### Nano-LC–MS

Nano-LC–MS/MS analyses were performed on an EASY-nLC™ HPLC system coupled to an Orbitrap Fusion™ Lumos™ mass spectrometer (Thermo Fisher Scientific). Half of each fraction was loaded onto a reversed-phase PepMap™ RSLC C18 column with Easy-Spray tip at 400 nL/min (ES802A, 2 μm, 100 Å, 75 μm × 25 cm). Peptides were eluted from 4 to 33% B over 120 min, 33–80% B over 5 min, and dropping from 50 to 10%B over the final 4 min (Mobile phases A: 0.1% FA, water; B: 0.1% FA, 80% Acetonitrile). Mass spectrometer settings include a capillary temperature of 300 °C and ion spray voltage was kept at 1.9 kV. The mass spectrometer method was operated in positive ion mode with a 4-s cycle time data-dependent acquisition with advanced peak determination and Easy-IC on (internal calibrant). Precursor scans (m/z 375-1600) were done with an orbitrap resolution of 120,000, 30% RF lens, 105 ms maximum inject time (IT), standard automatic gain control (AGC) target. MS2 filters included an intensity threshold of 2.5e−4, charges states of 2 to 6, 70% precursor fit threshold, and 60 s dynamic exclusion with a dependent scan being performed on only one charge state per precursor. Higher-energy collisional dissociation (HCD) MS2 scans were performed at 50 k orbitrap resolution, fixed collision energy of 37%, 200% normalized AGC target, and dynamic maximum IT.

### MS data analysis

Resulting RAW files were analyzed in Proteome Discover 2.4 (Thermo Fisher Scientific) with FASTA databases including Swiss-Prot UniProt Homo sapiens sequences (downloaded 09/17/2019) plus common contaminants. SEQUEST HT searches were conducted with a maximum number of 2 missed cleavages; precursor mass tolerance of 10 ppm; and a fragment mass tolerance of 0.02 Da. Static modifications used for the search were, (1) carbamidomethylation on cysteine (C) residues; (2) TMT sixplex label on lysine (K) residues and the N-termini of peptides (for TMT quant samples only). Dynamic modifications used for the search were oxidation of M, phosphorylation on S, T, Y, and acetylation of N-termini. IP-MS Sequest results were imported into Scaffold (Proteome Software) for Fishers exact test comparison. TMT quantification methods utilized isotopic impurity levels available from Thermo Fisher. Percolator False Discovery Rate was set to a strict setting of 0.01 and a relaxed setting of 0.05. Values from both unique and razor peptides were used for quantification. In the consensus workflow, peptides were normalized by total peptide amount with no scaling. Resulting abundance values for each sample, and abundance ratio values from Proteome Discoverer™ were exported to Microsoft Excel.

### Whole mRNA sequencing and data analyses

Total RNAs were extracted by using the RNeasy kit (Qiagen), and mRNA was enriched and sequenced by using paired-end deep-transcriptome sequencing with the Illumina platform at the IU Genomic Core. All mRNAs were profiled according to their FPKM values. The RNA-seq data were collected and analyzed as we previously described [21, 26]. Briefly, quality control for raw mRNA-seq data was generated by FastQC v0.11.5 (http://www.bioinformatics.babraham.ac.uk/projects/fastqc/). Illumina adapter sequences and low-quality bases were trimmed by Trim Galore v0.4.5 (http://www), followed by sequence mapping of high-quality paired-end reads to the human genome (hg38) with the aligner STAR v2.7.2b [29]. We further used bam-filter in ngsutilsj v0.4.8 (https://compgen.io/ngsutilsj) to keep only properly and uniquely mapped paired reads (MAPQ ≥ 10) for downstream analysis. FeatureCounts from package subread v1.6.5 [30] was employed to summarize gene expression levels based on mapped reads according to GENECODE v31 annotation. Analysis of differential expression genes (DEGs) was performed by edgeR v3.32.1 [31], with read counts normalized by the trimmed mean of *M*-values (TMM) method after lowly expressed genes filtered out by the filterByExpr function using default settings. DEGs due to overexpression of SARS-CoV-2 viral coding genes were identified if their FDR-adjusted *p* values were less than 0.05 based on the comparison between the viral gene overexpression and the control.

### Functional enrichment analysis

The functional enrichment analysis of gene ontology (GO) biological process was carried out by using DAVID (https://david.ncifcrf.gov/), THE GENE ONTOLOGY RESOURCE (http://geneontology.org/) and Gorilla (http://cbl-gorilla.cs.technion.ac.il/). Canonical signaling pathway and toxicity analysis were performed on QIAGEN Ingenuity Pathway Analysis (IPA).

### Protein–protein interaction (PPI) analysis

Protein–protein Interaction Networks analysis was carried out using the STRING software (https://string-db.org/).

### Quantification and statistical analysis

Data comparisons between two groups were made using an unpaired two-tailed *t* test. All data were presented as mean ± S.D. from at least three independent experiments. Differences with *P* values less than 0.05 were considered significant.

## Results

### Prediction of SARS-CoV-2 coding genes that potentially affect functions of human cardiomyocytes (CMs)

**Fig. 1:**
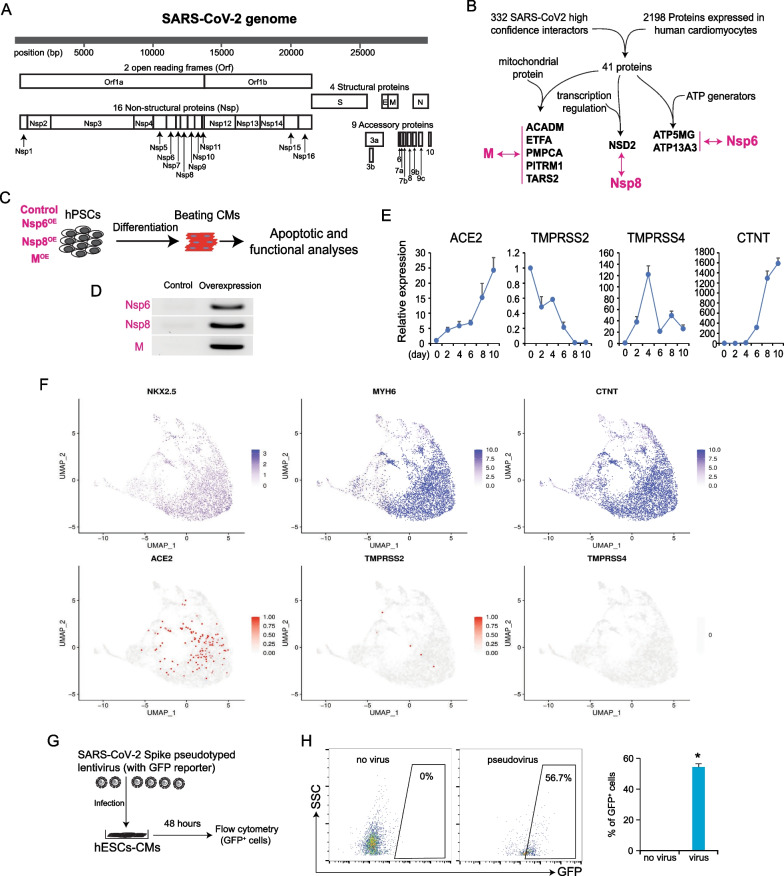
Prediction of harmful SARS-CoV-2 viral genes for human cardiomyocytes. **A** Coding genes annotation of the SARS-CoV-2 genome. **B** Strategies to predict the potential interactions between SARS-CoV-2 encoded proteins and essential proteins highly expressed in human cardiomyocytes. **C** Stable hPSC cell lines with overexpression of 3 SARS-CoV-2 genes by lentivirus were established and followed with CM differentiation and functional analyses. OE, overexpression. **D** RT-PCR detection of Nsp6, Nsp8, and M expression levels in hESC-derived CMs. **E** RT-qPCR detection of ACE2, TMPRSS2, TMPRSS4 and the cardiomyocyte marker CTNT during cardiac differentiation from hESCs. RNA samples were collected every 2 days from day 0 to day 10 of differentiation. Beating CMs were observed on day 8. All dots are shown as mean ± SD (*n* = 3). **F** Gene expression profiles of SARS-CoV-2 viral receptor genes ACE2, TMPRSS2/4, and cardiomyocyte markers NKX2.5, MYH6, CTNT in hESC-derived cardiomyocytes based on our previously published scRNA-seq data [34]. **G** SARS-CoV-2 Spike pseudotyped lentivirus carrying a GFP reporter was used to infect hESC-CMs. **H** Flow cytometry analysis of GFP^+^ hESC-CMs after infection with SARS-CoV-2 Spike pseudotyped lentivirus carrying a GFP reporter. 48 h post-infection, flow cytometry was performed to detect GFP^+^ cells. CMs without virus infection served as control. All bars are shown as mean ± SD. (*n* = 3). A two-tailed unpaired *t* test was used to calculate *p* values. *p < 0.05 (vs. no virus)

Myocardial injury and dysfunction are commonly observed in patients infected by SARS-CoV-2 virus [32]. The 30 kb SARS-CoV-2 viral genome encodes up to 27 genes (Fig. 1A). Currently, the responsive mechanisms of human heart muscle cells to individual SARS-CoV-2 viral genes remain elusive. To predict potential harmful SARS-CoV-2 genes for human CMs, we reanalyzed a previously published dataset [33], which established the interactome of each SARS-CoV-2 gene in human HEK-293T/17 cells. By comparing 332 high-confidence SARS-CoV-2 interactors in the HEK-293T/17 cells with 2198 proteins highly expressed in human CMs (human protein atlas, http://www.proteinatlas.org), we identified three SARS-CoV-2 coding genes, Nsp6, M and Nsp8, that could selectively interact with proteins vital for human CM survival and energy production (Fig. 1B). Subsequently, we undertook functional studies of these three genes in hPSC-CMs. Human H9 ES cells and wild type (WT) human iPS cells were transducted with lentivirus to overexpress M, Nsp8, or Nsp6, respectively, followed by differentiation into contracting CMs (Fig. 1C). This approach ensured consistently high expression levels of these SARS-CoV-2 genes in fully differentiated hESC-CMs (Fig. 1D). Considering that the SARS-CoV-2 virus invades human tissue cells through ACE2, TMPRSS2, or TMPRSS4 binding, we tracked their expression dynamics during CM differentiation from hESCs (Fig. 1E). Cardiac troponin T (cTNT) is a marker of CMs (Fig. 1E). ACE2 expression rapidly increased during CM differentiation, showing an over 20-fold increase in hESC-derived CMs (hESC-CMs) compared to hESCs. Conversely, TMPRSS2 expression rapidly declined during CM differentiation, while TMPRSS4 expression fluctuated (Fig. 1E). Reanalysis of our previously published scRNA-seq data [34] confirmed enhanced ACE2 expression over TMPRSS2 or TMPRSS4 in hESC-CMs, which co-expressed CM marker genes NKX2.5, MYH6, and CTNT (Fig. 1F). Additionally, adult human heart tissue expressed higher levels of ACE2 than TMPRSS2 and TMPRSS4, suggesting that SARS-CoV-2 primarily infects human CMs via ACE2 (Additional file 1: Fig. S1A-C). Finally, we introduced a pseudotyped SARS-CoV-2 virus carrying a GFP reporter into WT hESC-CM culture and observed that over 50% of hESC-CMs were GFP positive (Fig. 1G, H), aligning with recent reports of direct hPSC-CM infection by SARS-CoV-2 virus [17, 18].

### Nsp6, Nsp8, and M globally altered the transcriptome and proteome of hPSC-CMs

**Fig. 2:**
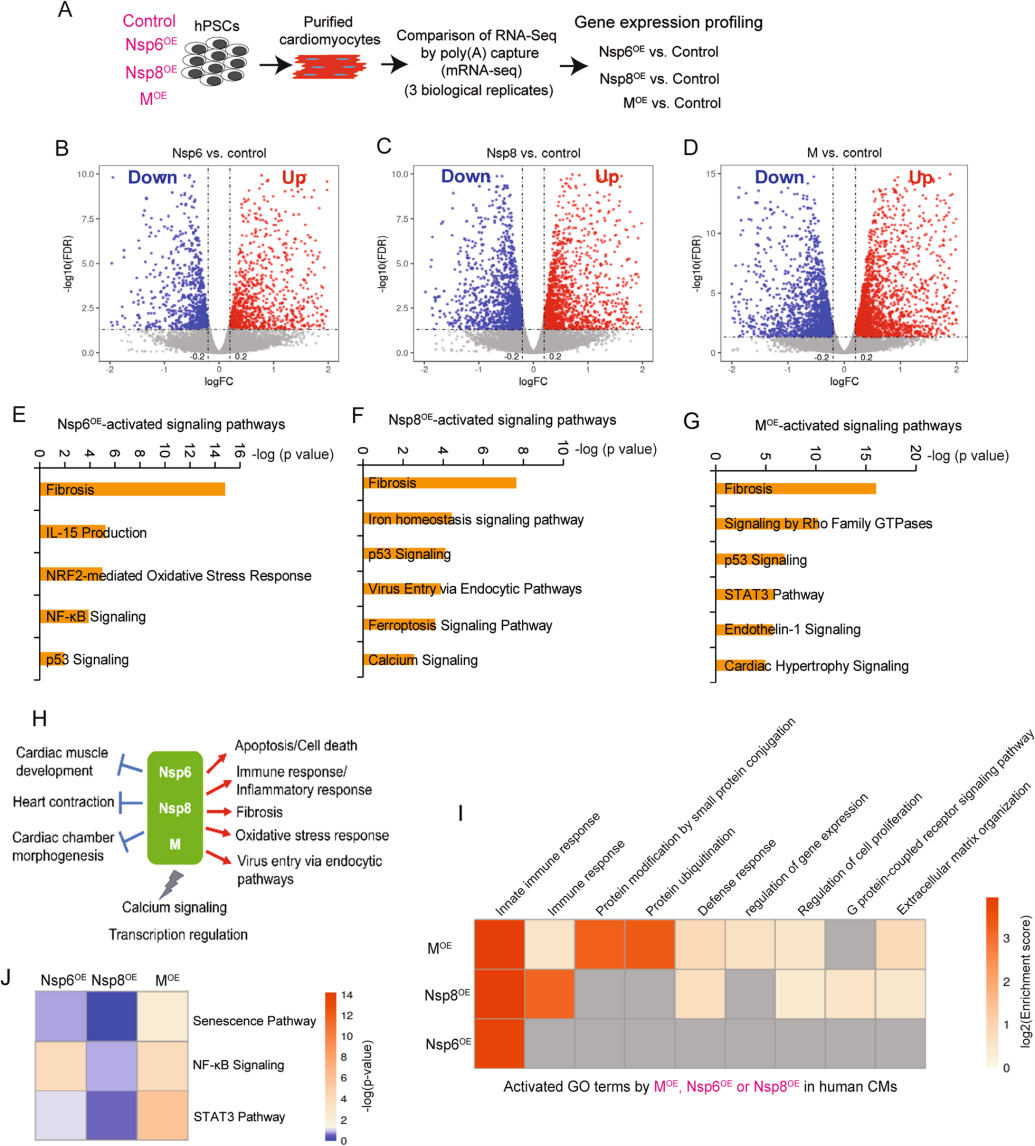
Whole mRNA-seq reveals the global impacts of SARS-CoV-2 viral genes on the transcriptome of hESC-CMs. **A** Scheme of whole mRNA-seq to study the global impacts of SARS-CoV-2 viral genes on the transcriptome of hESC-CMs. **B**–**D** Volcano blots showing differentially expressed genes caused by Nsp6^OE^ (**B**), Nsp8^OE^ (**C**), and M^OE^ (**D**) in hESC-CMs. **E**–**G** Canonical signaling pathways (SP) analyses of the upregulated genes induced by Nsp6^OE^ (**E**), Nsp8^OE^ (**F**) and M^OE^ (**G**) in hESC-CMs. **H** Summary of up- (red) or down-regulated (blue) DEGs by Nsp6^OE^, Nsp8^OE^ and M^OE^ in hESC-CMs. **I**–**J** Canonical signaling pathways of the genes that are differentially induced by Nsp6^OE^, Nsp8^OE^ and M^OE^ in hESC-CMs

Nsp6^OE^, Nsp8^OE^, or M^OE^ and control hESCs (OE, overexpression; Fig. 2A, Additional file 1: Fig. S1D) were differentiated into CMs, followed by CM enrichment to receive over 90% purity as previously described [35]. Next, the whole mRNA-seq was performed to profile differentially expressed genes (DEGs). Empty lentivector-infected hESCs were used as control. Compared to control hESC-CMs, each of the overexpressed SARS-CoV-2 genes significantly altered the global transcriptional profile of hESC-CMs (Fig. 2B–D, Additional file 2: Table S1). GO analysis of significantly altered genes (FDR < 0.05) highlighted that Nsp6^OE^, Nsp8^OE^, and M^OE^ co-activated genes associated with apoptosis/p53, immune response, inflammatory and oxidative stress response, fibrosis, and virus entry via endocytic pathways, whereas co-suppressed genes related to cardiac muscle development and contraction and calcium signaling (Fig. 2E–H, Additional file 1: Fig. S2A-E). Interestingly, each SARS-CoV-2 gene also differentially influenced the expression of genes enriched in signaling pathways, such as protein modifications, regulation of gene expression, senescence, NF-kB and STAT3 signaling pathways (Fig. 2I–J), suggesting the diverse impacts of individual SARS-CoV-2 genes on host hESC-CM transcriptome.

**Fig. 3:**
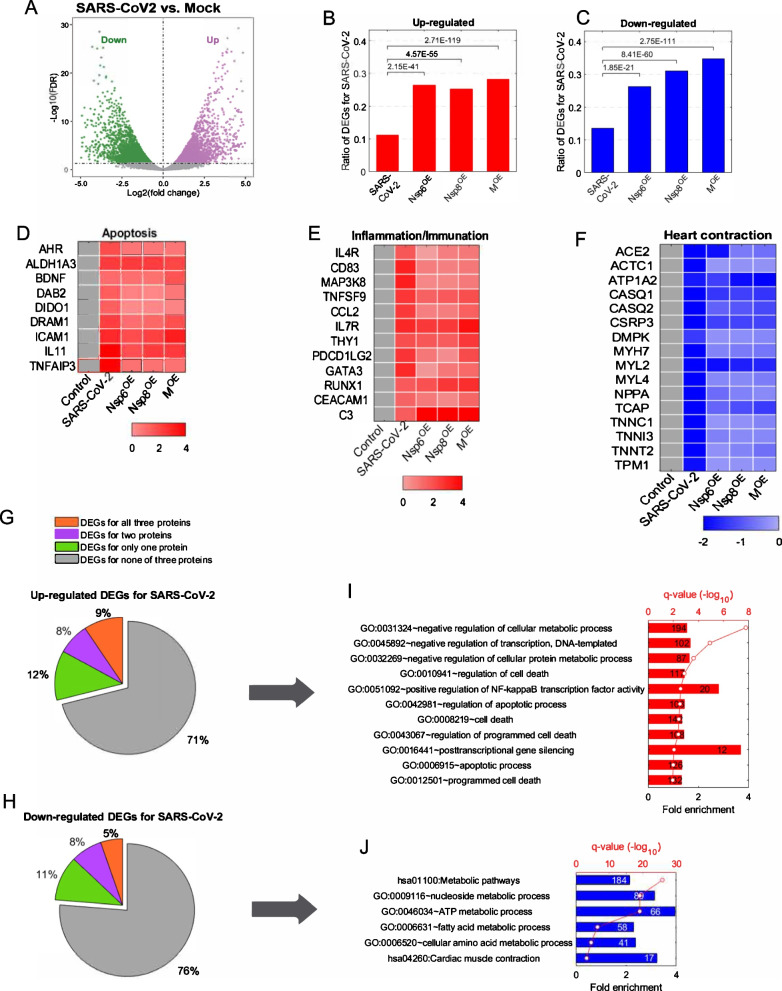
Concordant transcriptomic analysis. **A** Volcano blots showing differentially expressed genes in hiPSC-CMs infected by SARS-CoV-2 virus versus Mock control [17]. **B**, **C** Comparison of DEGs between hESC-CMs (Nsp6^OE^, Nsp8^OE^ and M^OE^ vs. Control) and hiPSC-CMs (SARS-CoV-2 vs. Mock). A two-tailed unpaired *t* test was used to calculate *p* values. The remarkable *p* value indicates the significant overlap between two gene sets. **D**–**F** Heat map showing representative apoptotic genes in **D**, inflammation genes in **E**, and heart contraction genes in **F** among indicated functional groups**. G** Frequencies of DEGs that are up-regulated by Nsp6^OE^/Nsp8^OE^/M^OE^ in DEGs that are up-regulated due to SARS-CoV-2 infection. **H** Frequencies of DEGs that are down-regulated by Nsp6^OE^/Nsp8^OE^/M^OE^ in DEGs that are down-regulated due to SARS-CoV-2 infection. **I** GO analysis of DEGs solely up-regulated by SARS-CoV-2 infection. **J** GO analysis of DEGs solely down-regulated by SARS-CoV-2 infection

Next, we conducted a concordant analysis to compare altered transcriptomic changes caused by the three SARS-CoV-2 genes to those induced by SARS-CoV-2 viral infection. Reanalysis of a previously published mRNA-seq dataset from SARS-CoV-2 infected hiPSC-CMs [17] revealed that both up- and down-regulated DEGs due to SARS-CoV-2 infection (Fig. 3A) were significantly enriched in DEGs resulting from Nsp6^OE^, Nsp8^OE^ and M^OE^ (Fig. 3B, C). The SARS-CoV-2 virus, Nsp6^OE^, Nsp8^OE^ and M^OE^ all enhanced the expression of genes related to apoptosis and inflammation/immune pathways, while repressing expression of genes in the heart contraction pathway (Fig. 3D–F). These findings indicate a concordance between the impacts of Nsp6^OE^, Nsp8^OE^ and M^OE^ and SARS-CoV-2 infection on hPSC-CM transcriptomic changes, suggesting these three SARS-CoV-2 genes play important roles in SARS-CoV-2-induced CM injuries. Interestingly, approximately 70% of DEGs (both up and down) in SARS-CoV-2-infected hiPSC-CMs were not significantly present in individual Nsp6^OE^, Nsp8^OE^, and M^OE^ hESC-CMs (Fig. 3G, H, grey areas), which were enriched into cellular metabolism, regulation of transcription, apoptosis (Fig. 3I), and ATP/fatty acid/amino acid metabolic processes (Fig. 3J). These differences indicate that other SARS-CoV-2 genes, such as Orf9c [21], could also alter the transcriptome of host hiPSC-CMs.

**Fig. 4:**
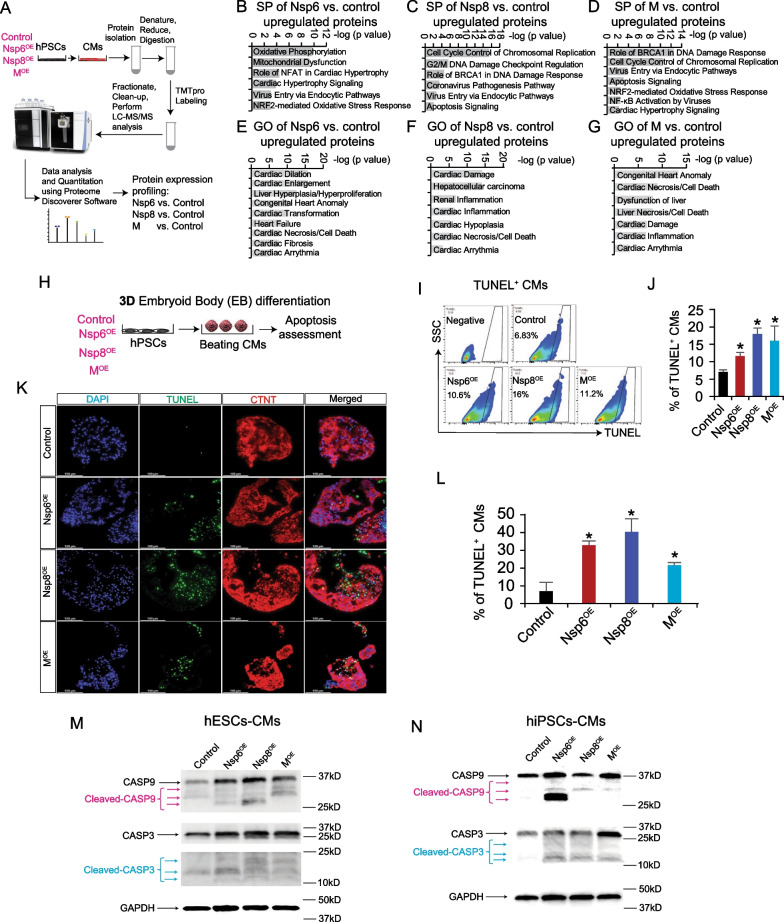
Overexpression of SARS-CoV-2 genes induces cell death of hPSCs-CMs. **A** The scheme showing CM differentiation from hESCs in 2D monolayer condition, followed by TMT-MS assessments. Wild type CMs serve as control. **B**–**D** Signaling pathway enrichment analysis of the upregulated proteins induced by Nsp6^OE^, Nsp8 ^OE^ and M ^OE^ in hESC-CMs. **E**–**G** GO analysis of the upregulated proteins induced by Nsp6^OE^, Nsp8^OE^ and M^OE^ in hESC-CMs. **H** The scheme of CM differentiation from hPSCs in 3D embryoid body (EB) condition, followed with apoptotic assessments. **I**–**J** Flow cytometry analysis of TUNEL^+^ cells in the CM (CTNT^+^) population of hiPSCs-derived EBs. All bars are shown as mean ± SD. (*n* = 4). A two-tailed unpaired *t* test was used to calculate *p* values: *p < 0.05 (vs. Control). **K** Representative immunostaining images of TUNEL^+^ cells in hiPSCs-derived EBs containing CTNT^+^ CMs. Scale bar, 100 µm. **L** Statistical data analysis of immunostaining results from (**K**). All bars are shown as mean ± SD. (*n* = 3). A two-tailed unpaired *t* test was used to calculate *p* values: *p < 0.05 (vs. Control). **M**–**N** Western blotting detection of CASP3, cleaved CASP3, CASP9 and cleaved CASP9 protein expressions in hESC-CMs (**M**) and hiPSC-CMs (**N**).

Our whole mRNA-seq results suggested that overexpression of SARS-CoV-2 viral genes could increase the expression of cell death/apoptosis-associated genes in hESC-CMs. To further explore this, we employed tandem mass tag-mass spectrometry (TMT-MS) to assess global protein expression changes in Nsp6^OE^, Nsp8^OE^, and M^OE^ versus control hESC-CMs (Fig. 4A, Additional file 3: Table S2). Consistent with mRNA-seq data, we observed an increase in proteins associated with stress-related apoptosis signal in the Nsp6^OE^, Nsp8^OE^, and M^OE^ hESC-CMs compared to control hESC-CMs (Fig. 4B–D). GO pathway enrichment analyses indicated that up-regulated proteins in Nsp6^OE^, Nsp8^OE^, and M^OE^ hESC-CMs contributed to cardiac fibrosis, cardiac arrhythmia, cardiac inflammation, and heart failure (4E–G).

### Nsp6, Nsp8, and M induce apoptosis of hPSC-CMs

We further evaluated apoptosis in Nsp6^OE^, Nsp8^OE^, M^OE^ hPSC-CMs using TUNEL assay. 3D beating embryoid bodies (EBs) were differentiated from hPSCs to mimic human myocardium-like tissues using our established differentiation method [25, 36] (Fig. 4H). Prominently increased percentages of TUNEL^+^/CTNT^+^ CMs were observed in Nsp6^OE^, Nsp8^OE^ or M^OE^ hiPSC-derived EBs compared to control hiPSC-EBs using flow cytometry and immunofluorescent staining (Fig. 4I–L). We also differentiated hESCs into CMs using a 2D monolayer differentiation method [24] (Additional file 1: Fig. S3A), finding a similar increase in the proportion of TUNEL^+^ CMs in Nsp6^OE^, Nsp8^OE^ and M^OE^ hESC-CMs (Additional file 1: Fig. S3B). Furthermore, we observed increased ratios of Annexin V^+^ CMs in Nsp6^OE^, Nsp8^OE^ or M^OE^ hESC-CMs compared to control hESC-CMs (Additional file 1: Fig. S3C-D). Annexin V has a strong affinity for phosphatidylserine (PS) residues on the surface of the cell and is an early marker of apoptosis. Finally, Western Blot analysis revealed elevated protein levels of two apoptotic markers, cleaved-CASP3 and cleaved-CASP9, in hESC-/hiPSC-EBs overexpressing Nsp6, Nsp8, or M, compared to control EBs (Fig. 4M, N, Additional file 1: Fig. S4A-B). Collectively, these results demonstrate that overexpression of Nsp6, Nsp8, or M can induce apoptosis in hPSC-CMs.

### Interactomes of Nsp6, Nsp8, and M in hPSC-CMs

**Fig. 5:**
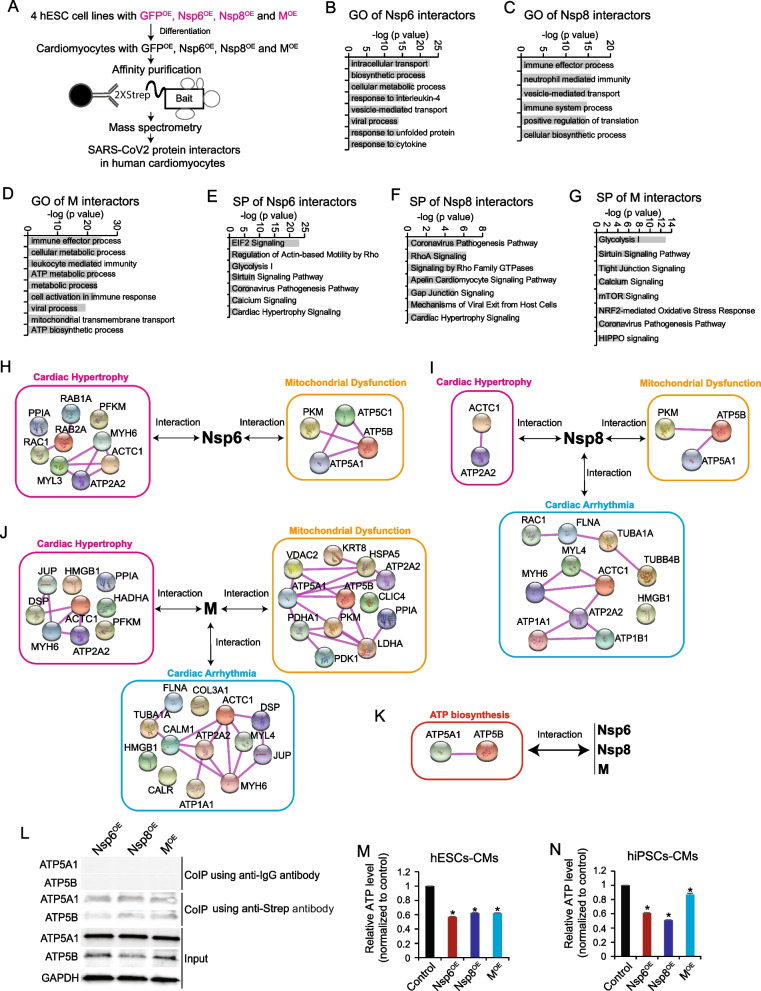
SARS-CoV-2 genes interact with host proteins in hPSC-CMs and reduce cellular ATP levels. **A** The scheme showing Co-IP MS method to globally study SARS-CoV-2 protein interactors in hESC-CMs. **B**–**D** GO analysis of Nsp6 interactors (**B**), Nsp8 interactors (**C**) and M interactors (**D**). **E**–**G** Signaling pathway (SP) analysis of Nsp6 interactors (**E**), Nsp8 interactors (**F**) and M interactors (**G**). **H** Protein–protein interaction (PPI) shows Nsp6 interactors are associated with cardiac hypertrophy and mitochondrial dysfunction. **I** Protein–protein interaction (PPI) shows Nsp8 interactors are associated with cardiac hypertrophy, mitochondrial dysfunction and cardiac arrhythmia. **J** Protein–protein interaction (PPI) shows M interactors are associated with cardiac hypertrophy, mitochondrial dysfunction and cardiac arrhythmia. **K** Protein–protein interaction (PPI) reveals the ATPase subunits ATP5A1 and ATP5B are shared interactors by Nsp6/Nsp8/M proteins in hESC-CMs. **L** Co-IP Western-blotting verification of the interactions of ATP5A1/ATP5B with Nsp6/Nsp8/M proteins in hESCs-CMs. **M** ATP level detection in hESC-CMs. All bars are shown as mean ± SD. (*n* = 3). A two-tailed unpaired *t* test was used to calculate *p* values. *p < 0.05 (vs. Control). **N** ATP level detection in hiPSC-CMs. All bars are shown as mean ± SD. (*n* = 3). A two-tailed unpaired *t* test was used to calculate *p* values. *p < 0.05 (vs. Control)

We generated four hESC lines that constitutively overexpressed Strep-tagged Nsp6, Nsp8, M, and GFP [33], thereby enabling the pull-down of all interacting proteins within host CMs. After the differentiation of hESCs into CMs, we performed co-immunoprecipitation mass spectrometry (Co-IP MS) to identify the interactome of Nsp6, Nsp8, and M in hESC-CMs (Fig. 5A). The complete list of interactors with Nsp6, Nsp8, and M proteins in hESC-CMs could be found in Additional file 4: Table S3. GO analyses of the pulled-down proteins revealed that Nsp6, Nsp8, and M all interacted with protein factors associated with immune response, viral process, and vesicle-mediated transport events (Fig. 5B–D). This finding aligns with a recent study in human HEK293T cells [37]. Furthermore, Nsp6, Nsp8, and M were found to interact with proteins involved in cellular metabolic processes, such as ATP biosynthesis and cellular biosynthetic processes (Fig. 5B–D), suggesting that these SARS-CoV-2 genes might disrupt the energy supply of human CMs. Enrichment analyses of canonical signaling pathways revealed that these genes could also interact with protein factors related to cell injury signaling pathways, such as the coronavirus pathogenesis pathway, viral exit from host cells, and calcium and cardiac hypertrophy signaling (Fig. 5E–G), which implies that these SARS-CoV-2 genes could induce comprehensive cellular injuries and cardiomyopathy in human CMs. Closer examination of the protein–protein interaction map and GO enrichment analyses revealed that the interactors of Nsp6, Nsp8, and M were all associated with cardiac hypertrophy and mitochondrial dysfunction (Fig. 5H). Further, interactors of Nsp8 (Fig. 5I) and M (Fig. 5J) were functionally related to cardiac arrhythmia. Importantly, all three genes interacted with ATPase subunits ATP5A1 and ATP5B in hESC-CMs (Fig. 5K), which was validated by Co-IP Western-blotting (Fig. 5L, Additional file 1: Fig. S4C). These findings strongly suggest that Nsp6, Nsp8, and M could impair ATP biosynthesis of hPSC-CMs. To confirm this, we quantified whole cellular ATP levels, and observed a significant reduction in ATP levels in both hESC-CMs and hiPSC-CMs overexpressing Nsp6, Nsp8, or M, compared with control CMs. Collectively, these results elucidate the interactomes of Nsp6, Nsp8, and M in hPSC-CMs, and underscore their detrimental roles in ATP homeostasis.

### Ivermectin and meclizine ameliorate apoptosis in Nsp6^OE^, Nsp8^OE^ and MOE hPSC-CMs

**Fig. 6:**
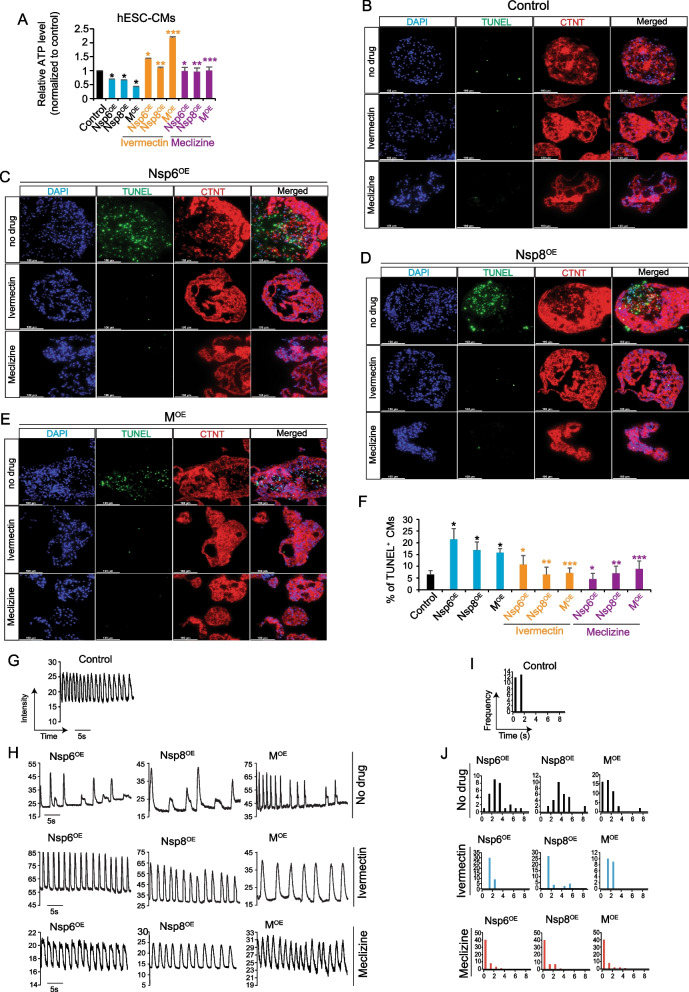
FDA-approved drugs can ameliorate SARS-CoV-2 genes-induced CM abnormalities. **A** ATP levels in hESC-derived CMs. The final concentration of ivermectin and meclizine's is 0.5 µM. Treatment time is 3 h. All bars are shown as mean ± SD. (*n* = 3). A two-tailed unpaired *t* test was used to calculate *p* values. *p < 0.05, **p < 0.01, ***p < 0.001. Black * indicates overexpression versus control; yellow * indicates ivermectin treatment versus no drug; purple * indicates meclizine versus no drug. **B**–**E** Representative immunostaining images of TUNEL^+^ and CTNT^+^ CMs in control hiPSC-EBs (**B**), Nsp6^OE^ hiPSC-EBs (**C**), Nsp8^OE^ hiPSC-EBs (**D**) and M^OE^ hiPSC-EBs (**E**). The final concentration of ivermectin and meclizine's is 0.5 µM. Treatment time is 48 h. Scale bar, 100 µm. **F** Statistical data analysis of immunostaining results from **B**–**E**. All bars are shown as mean ± SD (*n* = 3). A two-tailed unpaired *t* test was used to calculate *p* values. *p < 0.05, **p < 0.01, ***p < 0.001. Black * indicates overexpression versus control; yellow * indicates ivermectin treatment versus no drug; purple * indicates meclizine versus no drug. **G** Calcium imaging results in control hESC-CMs. *Y*-axis represents intensity and *X*-axis represents time (in seconds, s). **H** Calcium imaging results in Nsp6^OE^, Nsp8^OE^ and M^OE^ hESC-CMs treated without or with drugs for 1 h. *Y*-axis means intensity. *X*-axis represents time (in seconds, s). **I** Interspike interval (ISI) distribution analysis from **G**. *Y*-axis means beating frequency in a specific time window. *X*-axis means time (in seconds, s). **J** Interspike interval (ISI) distribution analysis from (**H**) in different CM cell lines treated with or without drugs for 1 h

Overexpression of Nsp6, Nsp8, or M was found to impair ATP production in hPSC-CMs. Given that reduced ATP levels are known to induce apoptosis in various cell types, including CMs [38–40], we hypothesized that pharmaceutical agents capable of enhancing cellular ATP biosynthesis might potentially counteract the apoptosis induced by Nsp6^OE^, Nsp8^OE^ or M^OE^ in hPSC-CMs. Previous studies have suggested that ivermectin (an antiparasitic drug) and meclizine (an antiemetic drug), both approved by the U.S. Food and Drug Administration (FDA), can protect mitochondrial function, and sustain cellular ATP levels [41–43]. Interestingly, after adding ivermectin (0.5 µM) or meclizine (0.5 µM) to culture media for 3 h, the ATP levels in Nsp6^OE^, Nsp8^OE^ and M^OE^ hESC-CMs significantly increased compared to the control groups without drug treatment (Fig. 6A). Notably, after 48 h of treatment with ivermectin (0.5 µM) or meclizine (0.5 µM), the ratios of TUNEL^+^ CMs in Nsp6^OE^, Nsp8^OE^ and M^OE^ hiPSC-EBs significantly decreased compared to control hiPSC-EBs without drug treatment (Fig. 6B–F). All these results demonstrate that Nsp6, Nsp8, and M can compromise cellular ATP production to induce apoptosis of hPSC-CMs, which can be ameliorated by ivermectin and meclizine through the restoration of cellular ATP levels.

### Ivermectin and meclizine mitigate Nsp6, Nsp8, and M-induced abnormal calcium handling in hPSC-CMs

ATP homeostasis is crucial for heart function [44, 45], and insufficient ATP production can impair intracellular Ca^2+^ signaling and excitation–contraction coupling in CMs to compromise their contractility [46–48]. We recorded electrically evoked [Ca^2+^]_i_ transients in X-Rhod-1-loaded Nsp6^OE^, Nsp8^OE^ and M^OE^ hESC-CMs using microfluorimetry. Representative time plots of changes in [Ca^2+^]_i_ were shown in Fig. 6G, H. Control hESC-CMs exhibited regular [Ca^2+^]_i_ transients with uniform amplitudes, while [Ca^2+^]_i_ transients of Nsp6^OE^, Nsp8^OE^ or M^OE^ hESÇ-CMs exhibited slower and irregular rates, along with the development of delayed aftertransients, which indicate increased Ca^2+^ leakage from the sarcoplasmic reticulum (SR). However, adding ivermectin (0.5 µM) or meclizine (0.5 µM) for 1 h prominently reduced the irregular rate and rhythmicity of spontaneous [Ca^2+^]_i_ transients and abrogated aftertransients in the Nsp6^OE^, Nsp8^OE^ or M^OE^ hESC-CMs. Additionally, histograms analysis of the interspike intervals (ISIs) generated from [Ca^2+^]_i_ transient recordings revealed a larger range of variations in the ISI distribution from the Nsp6^OE^, Nsp8^OE^ or M^OE^ hESC-CMs than control hESC-CMs, which could be notably reduced following treatment with ivermectin or meclizine (Fig. 6I–J). These results indicate that ivermectin and meclizine may mitigate the abnormal calcium handling induced by Nsp6, Nsp8, and M in hPSC-CMs.

## Discussion

Cardiac manifestations due to SARS-CoV-2 infection are of paramount importance for managing both acute and post-acute COVID-19 patients [9, 49, 50]. This study uncovers the genome-wide repercussions of SARS-CoV-2 viral genes Nsp6, Nsp8, and M on human pluripotent stem cell-derived cardiomyocytes, providing critical insights into the molecular pathogenesis of COVID-19 related cardiac dysfunction. We further demonstrate that the FDA-approved drugs, ivermectin and meclizine, can enhance cellular ATP levels, which in turn mitigated the induced apoptosis and functional abnormalities in hPSC-CMs. In summary, our study offers valuable insights into the detrimental effects of SARS-CoV-2 genes, Nsp6, Nsp8, and M, on the survival and function of human CMs, as well as the therapeutic interventions to protect cardiac function in COVID-19 patients.

SARS-CoV-2 infection contributes to a high risk of cardiovascular diseases even 1-year post-infection, irrespective of patient age or gender [9, 10]. In this study, we highlight the susceptibility of hPSC-CMs to three SARS-CoV-2 genes: Nsp6, Nsp8, and M. Nsp6 and Nsp8 are non-structural proteins, while M is the most abundant structural protein in SARS-CoV-2 virus. Our findings indicate that the enforced expression of these genes induces apoptosis and dysfunction in hPSC-CMs, suggesting their potential detrimental impacts on other SARS-CoV-2 target organs/tissues. This is consistent with a previous study showing that Nsp6 overexpression in fly hearts and mouse cardiomyocytes increases glycolysis and hypertrophic marker gene expression, leading to heart failure [51]. Moreover, Nsp6 has been shown to induce inflammatory cell death in lung epithelial cells [52], while the SARS-CoV-2M protein has a proapoptotic effect on human lung epithelial cells and umbilical vein cells [53]. Interestingly, while Nsp6, Nsp8, and M overexpression and SARS-CoV-2 infection similarly altered the hPSC-CMs transcriptome (Fig. 3B, C), about 70% of differentially expressed genes were exclusively influenced by SARS-CoV-2 (Fig. 3G, H), suggesting that the other 24 SARS-CoV-2 genes may also modify the host human CMs' transcriptome through different mechanisms. We reported that overexpression of Orf9c, a non-structural accessory protein of SARS-CoV-2, induces apoptosis and dysfunction of hPSC-CMs [21]. Orf9c overexpression and SARS-CoV-2 infection have concordant impacts on the hPSC-CMs transcriptome, affecting key signaling pathways for survival, function, and inflammation in human CMs. Therefore, our results suggest that among the 27 SARS-CoV-2 viral genes, Nsp6, Nsp8, M, and Orf9c may significantly contribute to the detrimental effects of SARS-CoV-2 virus on human heart muscle cells. However, it is notable that these SARS-CoV-2 genes are overexpressed in hiPSC-CMs at a very high expression level, which is different from SARS-CoV-2 virus infected hiPSC-CMs. Such expression dose difference might also contribute to the differential transcriptomic and proteomic changes between Nsp6, Nsp8, and M overexpressing hiPSC-CMs and SARS-CoV-2 virus infected hiPSC-CMs.

In this study, we found that Nsp6, Nsp8, and M all interacted with ATPase subunits ATP5A1 and ATP5B, thereby compromising the cellular ATP levels of hPSC-CMs (Fig. 5K–N). Similarly, Nsp6 directly interacted with ATP6AP1, a component of the vacuolar ATPase proton pump, in lung epithelial cells [52], and M protein triggers mitochondrial apoptosis via the BOK protein [53]. Therefore, our findings suggest a central role of ATP homeostasis in SARS-CoV-2-induced tissue injuries in CMs, and likely in other SARS-CoV-2 target cells in the lung and kidney. However, the precise mechanisms through which Nsp6, Nsp8, and M hijack ATPase require further investigation. Additionally, apoptosis and imbalanced calcium handling in Nsp6^OE^, Nsp8^OE^ or M^OE^ hPSC-CMs could also lead to intracellular ATP reduction.

We explored pharmaceutical interventions to enhance cellular ATP levels and found that two FDA-approved drugs, ivermectin and meclizine, significantly ameliorated Nsp6, Nsp8, and M-induced cell death and calcium dysfunction in human CMs. Although ivermectin is primarily used for treating parasitic infections, it has been identified as a mitochondrial ATP protector in CMs, enhancing mitochondrial ATP production in HL-1 CMs by upregulating the transcription of Cox6a2, a subunit of the mitochondrial respiratory chain [41]. Meclizine, on the other hand, exhibits cardio-protective effects by promoting CMs' glycolysis [43], thereby increasing ATP synthesis, mitigating ATP depletion, and protecting mitochondrial function [54]. These studies corroborate our findings that ivermectin and meclizine may increase ATP levels to mitigate the cell death in hPSC-CMs induced by SARS-CoV-2 genes.

## Conclusions

In this study, we comprehensive reveal the global detrimental impacts of SARS-CoV-2 genes Nsp6, Nsp8, and M on hPSC-CMs. We found that overexpression of Nsp6, Nsp8, and M disrupted the transcriptome and proteome of hPSC-CMs. Importantly, Nsp6, Nsp8, and M interact with ATPase subunits ATP5A1/ATP5B, leading to a reduction in ATP levels in hPSC-CMs, which in turn precipitates CM death and functional abnormalities. We identified that FDA-approved drugs, ivermectin and meclizine, could enhance cellular ATP levels and attenuate the Nsp6, Nsp8, and M-induced cell death and abnormal calcium handling in hPSC-CMs. Collectively, our findings suggest that preserving ATP homeostasis may represent a crucial therapeutic strategy for addressing COVID-19-induced cardiac injuries and functional abnormalities.

### Supplementary Information

**Additional file 1.** Supplementary Figures.

**Additional file 2. Table S1.** mRNA expression profiling of Nsp6OE, Nsp8OE, or MOE and control hESC-CMs.

**Additional file 3. Table S2.** Protein expression profiling of Nsp6OE, Nsp8OE, or MOE and control hESC-CMs.

**Additional file 4. Table S3.** CoIP-MS of Nsp6OE, Nsp8OE, or MOE and control hESC-CMs.

**Additional file 5. Table S4.** Primers for RT-qPCR.

## Data Availability

The GEO accession number for the mRNA-seq dataset is GSE171742.

## Abbreviations

SARS-CoV-2: Severe acute respiratory syndrome-coronavirus-2
COVID-19: Coronavirus disease-2019
ACE2: Angiotensin converting enzyme 2
hPSCs: Human pluripotent stem cells
