# Supplementary material for: SARS-CoV-2 viral genes Nsp6, Nsp8, and M compromise cellular ATP levels to impair survival and function of human pluripotent stem cell-derived cardiomyocytes
Source: Stem Cell Res Ther. 2023 Sep 13;14:249. doi: 10.1186/s13287-023-03485-3 (PMC10500938; doi:10.1186/s13287-023-03485-3)

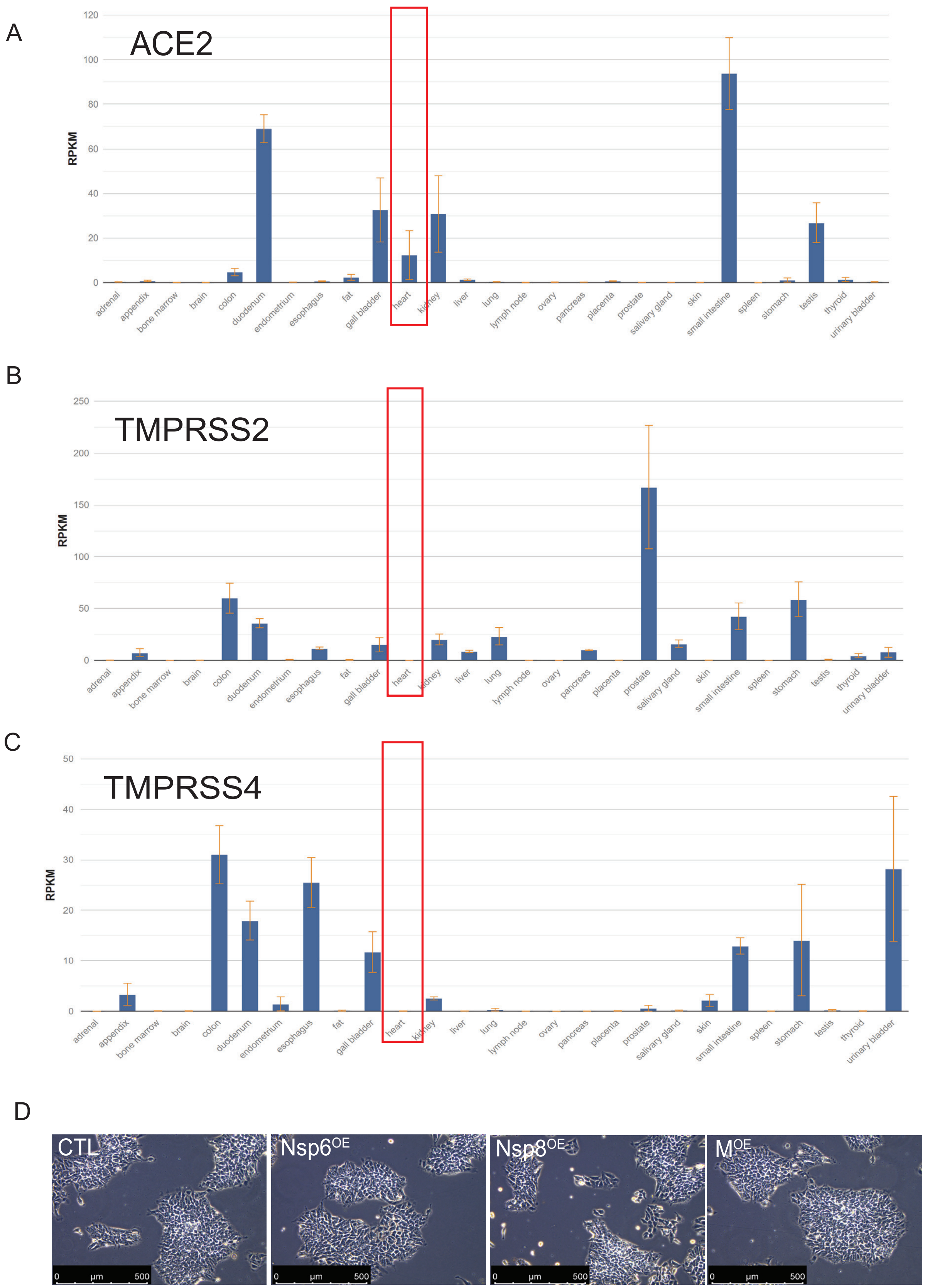

Supplementary Figure S1.Expression of SARS-Cov-2 receptor genes in the human heart.

(A) RNA expression level of ACE2 gene in adult human heart tissues from RNA-seq data (NCBI database).
(B) RNA expression level of TMPRSS2 gene in adult human heart tissues from RNA-seq data (NCBI database).
(C) RNA expression level of TMPRSS4 gene in adult human heart tissues from RNA-seq data (NCBI database).
(D) Representative images of establsihed control and Nsp6^{OE}, Nsp8^{OE} and M^{OE} hESC lines.

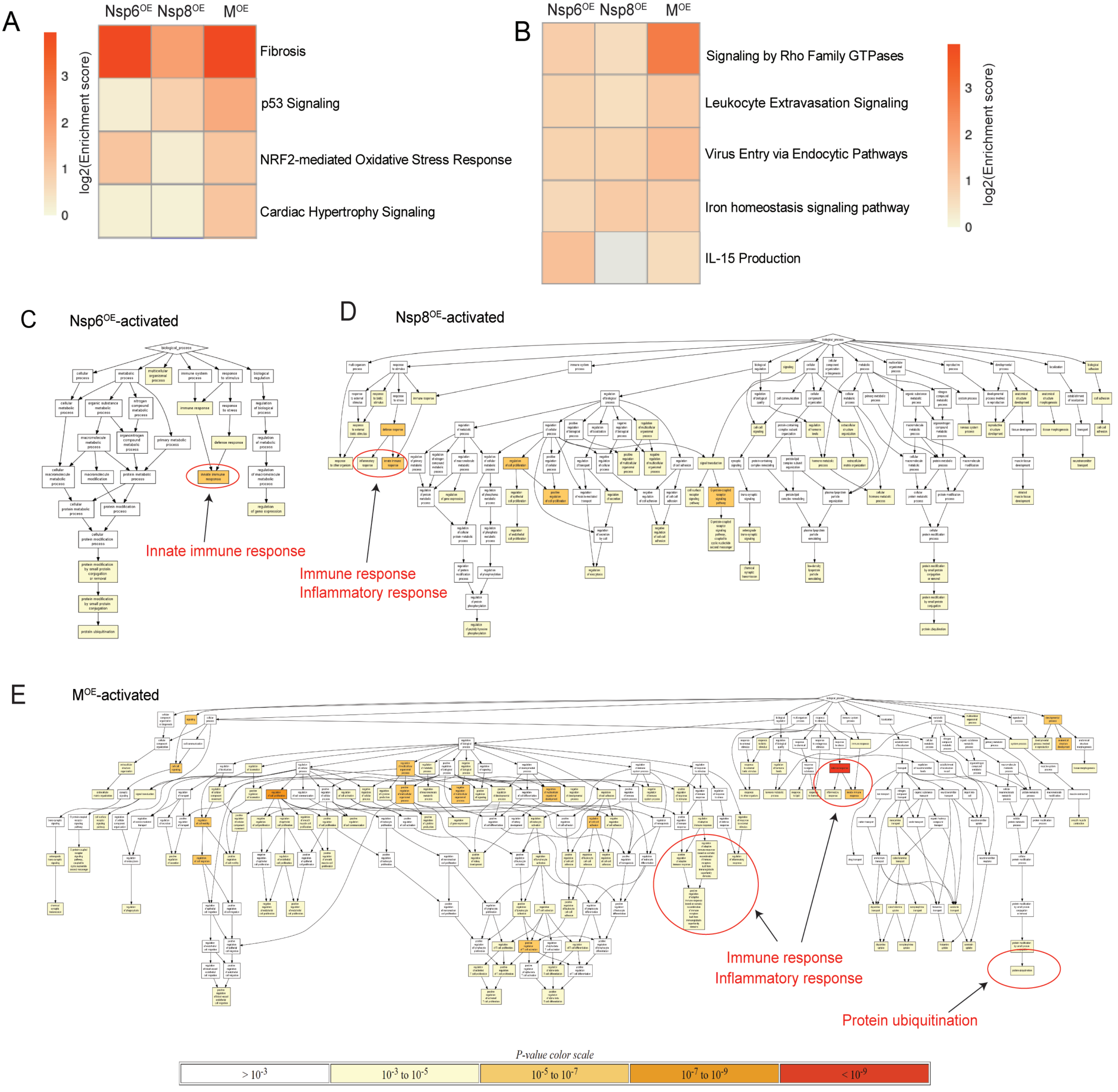

Supplementary Figure S2. GO analysis of DEGs in hESC-CMs.

(A-B) Signaling pathways activated by Nsp6^{OE}, Nsp8^{OE} and M^{OE} in hESC-CMs.

(C-E) GO hierarchy activated by Nsp6^{OE}, Nsp8^{OE} and M^{OE} in hESC-CMs.

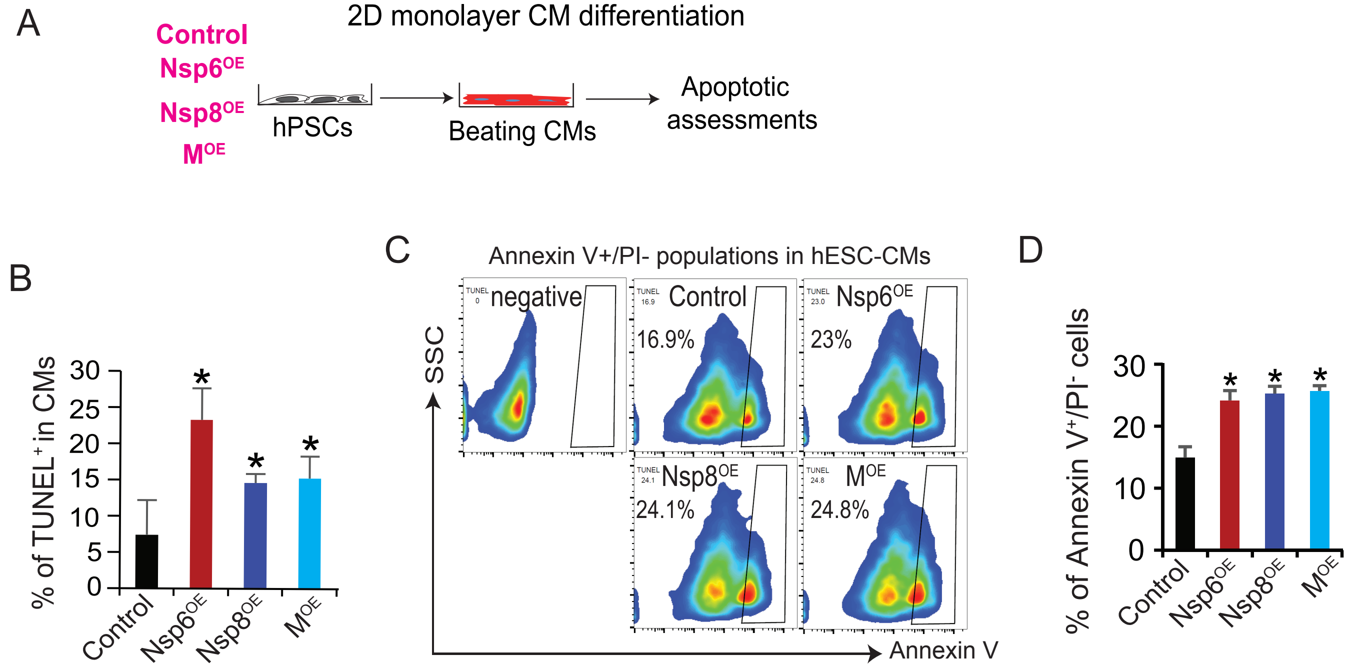

Supplementary Figure S3. Overexpression of SARS-CoV-2 genes induces cell death in hPSC-CMs.
(A) Scheme of cardiomyocyte differentiation from hESCs in 2D monolayer conditions, followed by apoptotic assessments.
(B) Flowcytometry analysis of TUNEL+ cells in hESC-CMs with overexpression of SARS-CoV-2 genes.
(C) Flowcytometry analysis of Annexin V+/PI- in hESC-CMs with overexpression of SARS-CoV-2 genes.
(D) Quantification results of (C).
All error bars show mean ± SD of three independent experiments. *p<0.05.(Student's *t*-test).

Supplementary Figure 4

A

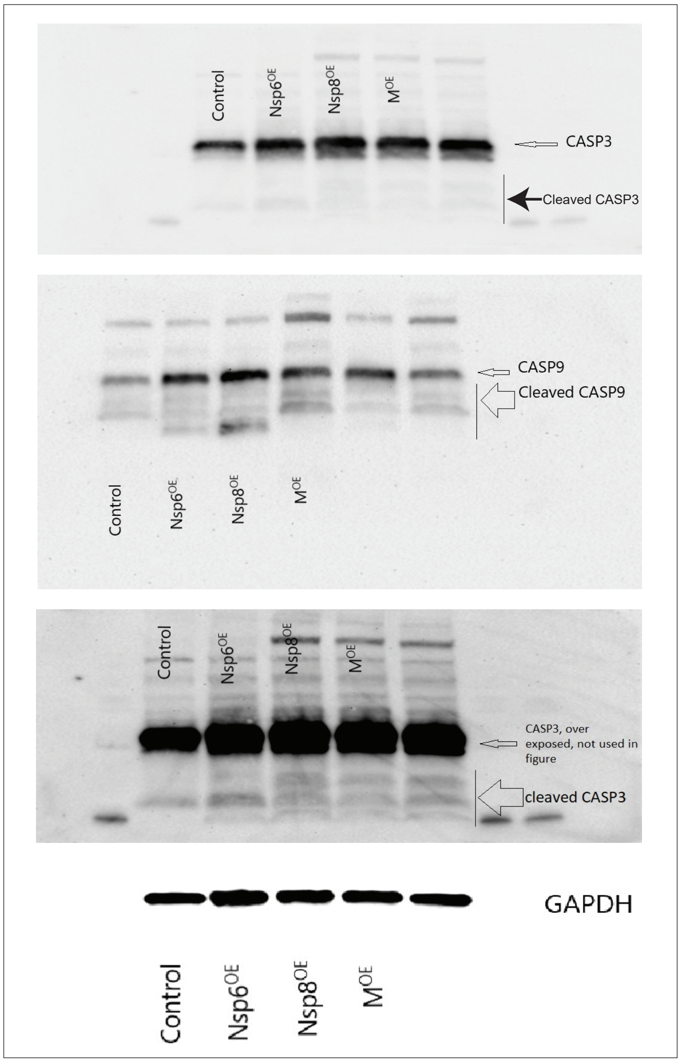

B

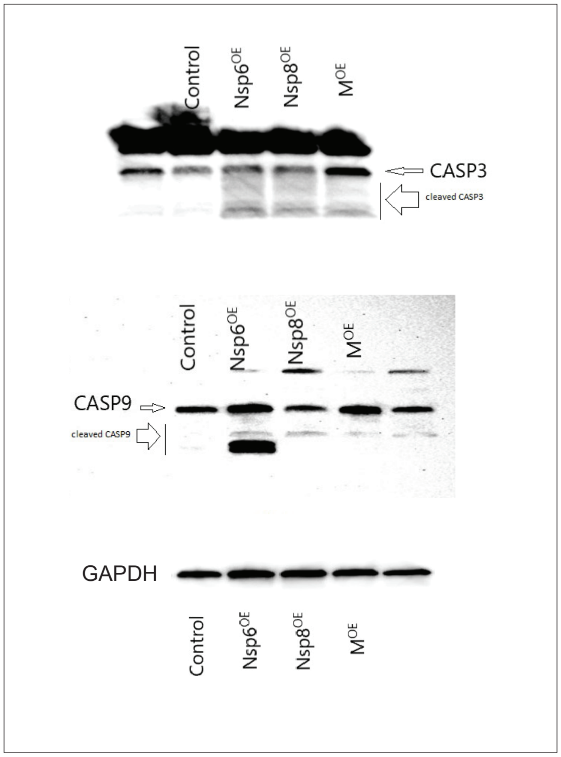

Supplementary Figure 4.
Original images of Western Blotting
for Figure 4M in (A), Figure 4N in (B) and Figure 5L in (C).

C

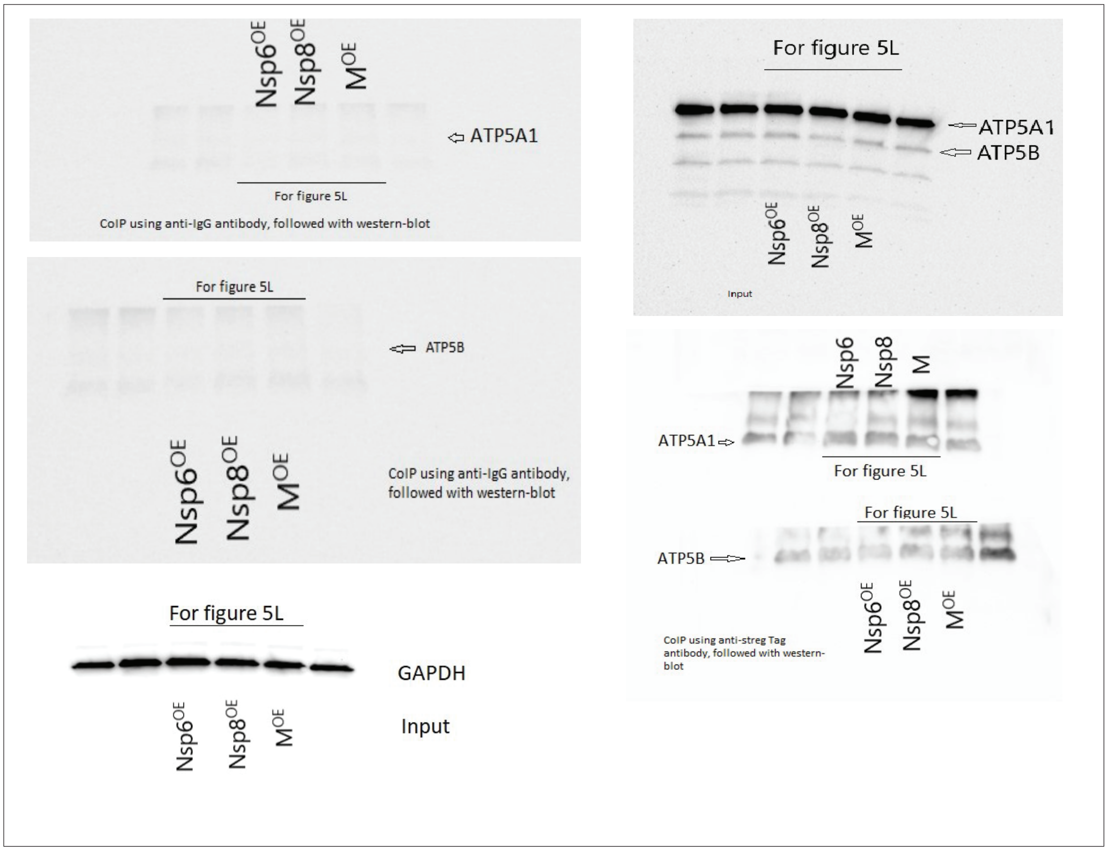

Supplement: Supplementary file 1 — Additional file 1. Supplementary Figures. [file 13287_2023_3485_MOESM1_ESM.pdf]
